# Total phenolic, anthocyanin contents and antioxidant capacity of selected elderberry (*Sambucus canadensis* L.) accessions

**DOI:** 10.4103/0973-1296.66936

**Published:** 2010

**Authors:** Mustafa Özgen, Joseph C. Scheerens, R. Neil Reese, Raymond A. Miller

**Affiliations:** Department of Horticulture, Gaziosmanpasa University, Tasliciftlik, Tokat- 60240, Turkey; 1 Department of Horticulture and Crop Science, The Ohio State University, Ohio Agricultural Research and Development Center, 1680 Madison Avenue, Wooster, OH 44691, USA; 2 Department of Biology and Microbiology, NPB 249B, Box 2140D, South Dakota State University, Brookings, SD 57007, USA

**Keywords:** DPPH, ferric reducing antioxidant power, small fruits, phytonutrient, radical scavenging assay

## Abstract

Fourteen purple-black American elderberry accessions (*Sambucus canadensis* L.) obtained from various sites in midwestern USA and then grown at a single Ohio production site in USA were analyzed for their total phenolic (TP) and total monomeric anthocyanin (TMA) contents and for their antioxidant capacity by the ferric reducing antioxidant power (FRAP) and DPPH radical scavenging assays. Total phenolic and anthocyanin contents were measured using the Folin-Ciocalteu reagent and the pH differential methods, respectively. Overall, the phytonutrient contents and antioxidant capacity of our elderberry accessions were similar to those typically reported for black raspberries, blackberries and other dark-fleshed small fruits. Variability among accessions was greatest for TMA content (CV 37.5%); individuals ranged nearly threefold from 1308 to 4004 *μ*g cy3-GE/g on a fresh weight basis. Variation among accessions was also evident for TP, FRAP and DPPH values (CV 14.4, 21.7 and 26.8%, respectively). TP and TMA values were very highly correlated (*r* = 0.93), although individuals differed in the estimated proportion of total phenolics attributable to anthocyanins. Both TP and TMA also highly correlated to antioxidant capacity values (*r* = 0.70–0.85). Within this limited study of 14 accessions, variability for phytonutrient content and antioxidant capacity suggested the employment of wild germplasm within an elderberry improvement program to incorporate an array of superior horticultural, post-harvest or processing traits into new or existing cultivars with superior phytonutrient profiles.

## INTRODUCTION

Small fruits are rich sources of both anthocyanins and phenolic compounds.[1–3] Anthocyanin pigments and other phenolic compounds function metabolically and/or ecologically in these fruits by providing a wide range of protective functions against predators or environmental stress and by serving as feeding attractants for birds or other seed dispersal agents.[4] When included in the human diet, they exhibit a wide range of antioxidant protection and therapeutic benefits including reduced risk of coronary heart disease, reduced risk of stroke, anticarcinogenic activity, improved visual acuity, and improved cognitive behavior.[5–9]

The purple-black fruits of elderberries (*Sambucus* spp. L.) are one of the richest sources of anthocyanins and phenolic compounds among small fruits and have strong antioxidant capacity.[10–12] Historically, elderberries have been used medicinally by many indigenous cultures. The health-promoting aspects of the fruit have received significant attention in the popular press, but scientific literature confirming the effects of elderberry and elderberry products on specific chronic diseases is less prevalent. Recently, extracts of both European, black or common elderberry (*Sambucus* nigra L.) and American elderberry (*Sambucus canadensis* L.) demonstrated significant chemopreventive potential controlling enzymes commonly associated with various forms of cancer.[13] European elderberry extracts have also demonstrated antioxidative, anti-inflammatory, immunostimulatory, and to a lesser degree, atheroprotective properties in pilot clinical or physiological trials.[14–16]

American elderberries are large, erect, stoloniferous, long-lived, perennial shrubs native of the complete eastern regions of North America.[17–19] These plants bloom in early summer, bearing complete flowers on large cymes produced laterally on perennial canes and terminally on new canes. Individual cymes can have hundreds of mature fruit, botanically described as berries which ripen from late summer to fall, depending on location. Individual berries are small (≈8 mm in diameter). They are edible as raw fruit but can be slightly bitter. Berries are most extensively processed for juices, jams, syrup, wines and pies.

Elderberry production is commercialized in Europe and products processed from raw fruit (i.e., foods, herbal supplements, industrial products) are available worldwide. Although a commercial industry is emerging in Oregon, USA,[11] American elderberries for human consumption are harvested predominantly from wild stands and then marketed or used locally. Cultivation of American elderberry was reported as early as 1761, but the first cultivars “Adams 1” and “Adams 2” were not released until 1926. Since then, very few additional cultivars have been developed. The principal health-promoting compounds of most American elderberry cultivars were well-characterized by Lee and Finn,[11] but variability in phytonutritional potential among wild germplasm is yet to be reported. Our objective was to determine ranges in fruit phytonutrient levels and antioxidant capacity within a small, predominantly midwestern segment of this germplasm pool. We anticipate these data may be potentially useful during the development of specific health-promoting cultivars if and when an optimal or target phenolic profile for elderberries emerges from medical and/or nutritional research.

## MATERIALS AND METHODS

### Plant materials

Fourteen feral American elderberry accessions, predominantly of midwestern origin, were collected and transplanted to a single Ohio production site over a period of years. Plants were chosen predominantly for their putative cultural potential, but they varied substantially in individual fruit size and other morphologic traits. Plants were cultivated at wide spacings, using recommended practices.[19] Fruits were harvested from these plants as they fully ripened. Harvested fruit samples were immediately frozen in 100 g batches; once frozen, they were transported on ice to the Ohio Agricultural Research and Development Center and stored at –20°C until analyzed.

### Analytical procedures

After thawing to room temperature, triplicate 100 g lots of elderberry fruits from each accession were homogenized in a blender. To ascertain and reference the maturity of samples, homogenates were analyzed for levels of total soluble solids (TSS) and titratable acidity (TA) by refractometry and standard titration methods as described by Perkins-Veazie and Collins.[20]

All triplicates were screened for their total phenolic (TP) contents, total monomeric anthocyanin levels (TMA) and antioxidant capacity, following a single extraction procedure.[21] For this procedure, a 3-g of aliquot of each homogenate was transferred to polypropylene tubes and extracted with 40 ml of extraction buffer containing acetone, deionized water and acetic acid (70:29.5:0.5 v/v) for 1 h. After filtration, the extracts were concentrated by rotary evaporation to remove the organic solvent and then each triplicate was readjusted to a concentration of 40 ml with deionized water. All the laboratory procedures were performed in duplicate from each triplicate extract.

Sample TP contents were measured according to the method of Singleton and Rossi[21] with slight modifications. To determine the levels of TP, 1 ml of each extract was combined with Folin-Ciocalteu’s phenol reagent and water 1:1:20 (v/v) and incubated for 8 min, followed by the addition of 10 ml of 7% (w/v) sodium carbonate. After 2 h, the absorbance of each was measured at 750 nm. Values of TP were estimated by comparing the absorbance of each with those of a standard response curve generated with gallic acid. Results are expressed as micrograms of gallic acid equivalents on a fresh weight basis (GAE/g fw).

TMA levels were measured by the pH differential method described by Giusti *et al*.[22] Sample extracts were combined in a 1:20 ratio (v:v) with potassium chloride and with sodium acetate buffers (pH 1.0 and 4.5, respectively) in separate vessels. After an equilibration period (15 min), the raw absorbance of each solution was measured at 533 and 700 nm. A corrected absorbance value was calculated as [(A520 – A700) pH 1.0 – (A520 – A700) pH 4.5]. The anthocyanin content was calculated using the molar absorptivity (є) and molecular weights (MW) of cyanidin 3-glucoside (є = 26,900; MW = 449.2). Results are expressed as micrograms of cyanidin 3-glucoside equivalents (Cy3-GE)/g fw.

As suggested in the literature,[23] two methods were used to determine total antioxidant capacity of samples. The ferric reducing antioxidant power (FRAP) method was conducted according to Benzie and Strain.[24] To conduct the assay, a 2.97-ml aliquot of FRAP reagent, a mixture of 0.1 mol/l acetate buffer, 10 mmol/l 2,4,6-Tris(2-pyridyl)-s-triazine (TPTZ) and 20 mmol/l ferric chloride (10:1:1 v/v/v) was combined with 30 μl of the extract. After incubation for 10 min, the absorbance of each solution was determined at 593 nm. The DPPH radical scavenging capacity was measured using the method of Brand-Williams *et al*.[25] The radical solution was prepared by dissolving DPPH (40 mg/l) in methanol. For the assay, a 2.97-ml aliquot of 2,2-diphenyl-1-picrylhydrazyl (DPPH) solution and 30 μl samples were mixed. After 10 min, the reaction absorbance was measured at 515 nm. To determine the antioxidant capacity of samples by both procedures, absorbance values were compared with those obtained from standard curves of trolox (10–100 μmol/l). Antioxidant capacity values were expressed as trolox equivalents (TE)/g fw.

### Data analysis

Data were analyzed using SAS procedures and software (SAS, Cary, NC, USA). Means, standard deviations, standard errors and coefficients of variation were obtained using PROC MEANS, whereas Pearson correlation coefficients and their probabilities were calculated using PROC CORR.

## RESULTS AND DISCUSSION

We found substantial variation in TMA content (coefficient of variation (CV) = 37.5%) among the 14 American elderberry accessions studied [Table 1]. Magnitude of difference was nearly threefold between the highest and the lowest anthocyanin content of the accessions (1308–4004 μg Cy3GE/g fw). Potentially useful variation in TP content and antioxidant capacity was also uncovered within our population. Our values for TP and TMA, their overall range and extent of variability within our accession population closely resembled those reported for eight American elderberry cultivars by Lee and Finn,[11] especially those determined for the second season of their study. Our accession values for TP, TMA and FRAP were also similar to those found in fruit juice samples by Byers and colleagues during an ongoing, multisite study quantifying medicinal compounds in fruit and other elderberry tissues (P.L. Byers, personal communication). In our study, Accession 13 displayed the lowest TP and TMA levels (2898 μg GAE/g fw and 1308 μg Cy3GE/g fw, respectively). Accessions 5 and 10 displayed exceptionally high levels of both TP (4583 and 5006 μg GAE/g fw, respectively) and TMA (4004 and 3833 μg Cy3GE/g fw, respectively).

**Table 1:** TMA, TP, antioxidant capacity (FRAP and DPPH), TA and TSS of elderberry genotypes

Accession	TP (μg GAE/g fw)	TMA (μg Cy−3GE/g fw)	FRAP (μmol TE/g fw)	DPPH (μmol TE/g fw)	TA (%)	TSS (%)
1	3889 ± 112	2557 ± 21	28.4 ± 0.3	12.4 ± 0.3	0.73 ±0.02	11.3 ± 0.1
2	3349 ± 59	1407 ± 38	22.3 ± 1.9	8.8 ± 0.5	0.76 ± 0.01	11.9 ± 0.1
3	3551± 26	1552 ± 29	23.9 ± 0.2	10.3 ± 0.1	0.65 ± 0.02	11.3 ± 0.1
4	3699± 218	2256 ± 158	24.9 ± 2.9	12.0 ± 1.2	0.84 ± 0.03	12.1 ± 0.5
5	4583 ± 24	4004 ± 93	31.7 ± 1.3	10.3 ± 0.5	0.84 ± 0.01	12.5 ± 0.6
6	3370 ± 41	1893 ± 59	20.8 ± 0.2	5.4 ± 0.6	0.61 ± 0.01	10.8 ± 0.4
7	4220 ± 91	2871 ± 67	27.3 ± 1.0	13.6 ± 0.7	0.95 ± 0.03	12.8 ± 0.4
8	3611 ± 106	1638 ± 96	20.0 ± 0.4	10.0 ± 1.0	0.91 ± 0.03	14.4 ± 0.1
9	3764 ± 78	1557 ± 47	17.5 ± 0.2	9.2 ± 0.4	0.93 ± 0.07	14.3 ± 0.2
10	5006 ± 64	3833 ± 45	30.6 ±1.5	16.9 ± 0.9	1.13 ± 0.03	12.9 ± 0.9
11	4072 ± 62	3090 ± 50	25.6 ± 1.0	12.1 ± 0.2	1.00 ± 0.05	10.8 ± 0.2
12	4535 ± 55	3343 ± 39	29.2 ± 1.2	13.5 ± 0.4	1.13 ± 0.03	12.2 ± 0.6
13	2898 ± 42	1308 ± 51	13.4 ± 1.1	7.0 ± 0.6	0.74 ± 0.04	7.9 ± 0.1
14	4014 ± 184	2675 ± 170	21.1 ± 2.7	10.3 ± 1.3	1.06 ± 0.04	10.0 ± 0.2
Grand mean	3897	2428	24.1	10.8	0.75	11.8
CV (%)	14.4	37.5	21.7	26.8	19.1	14.2

Accession values represent triplicate means ± standard errors from the mean; population variability is indicated by the grand mean and its associated coefficient of variability (i.e., the population standard deviation expressed as a percentage of the mean). TP content was estimated by the Folin-Ciocalteu assay of Singleton and Rossi.[21] Values are expressed as micrograms of gallic acid equivalents/g fw. TMAs were determined by the pH-differential method of Giusti and Wrolstad.[22] Values are expressed as micrograms of cyanidin 3-glucoside equivalents/g fw. FRAP values were determined by the method of Benzie and Strain.[24] Values are expressed as micrograms of trolox equivalents/g fw. DPPH values were determined by the method of Brand-Williams *et al*.[25] Values are expressed as micrograms of trolox equivalents/g fw. TA was determined by the titration method of Perkins-Veazie and Collins.[20] TSSs were determined by refractometry

Proportionally, anthocyanins appeared to be the predominant phenolic constituents in our accessions, and, as with other dark-fleshed fruits,[26,27] these two parameters are very highly related (*r*^2^ = 0.93, Table 2]. However, the relative proportion of anthocyanins to TP levels varied substantially among individual accessions, ranging from 41.4% to 87.9% (Accessions 9 and 5, respectively). Similar TMA/TP values were reported earlier among American elderberry cultivars.[11] Admittedly, our assessments of TP, TMA and their ratio were based upon the underlying assumptions that photocell response to a single anthocyanin moiety (Cy3G) and a single phenolic compound (GA) fairly represented that of the other compounds present. The American elderberry anthocyanin profile was reported to contain a complex mixture of cyanidin glycosides and, especially their acylated (coumaroyl) derivatives[11,28] and the profile of non-anthocyanin phenolics, yet to be fully elucidated, is also likely to be complex. Therefore, among our accessions, absolute quantities for TP, TMA and their ratio may have differed from our estimates. Nevertheless, as phenolic compound classes were presumed to be the predominant health promoting properties of elderberries,[13–15] the substantial variability among accessions, based on our estimates of TP, TMA and their ratio, may have particular dietary relevance.

**Table 2:** Correlation coefficients (r) of TP, TMA, antioxidant capacity (FRAP and DPPH), and TA as a maturity indicator

Source	TP	TMA	FRAP	DPPH	TA
TMA	0.93*				
FRAP	0.84*	0.85*			
DPPH	0.82*	0.70*	0.74*		
TA	0.73*	0.61*	0.35	0.71*	
TSS	0.43	0.16	0.33	0.37	0.32

* Indicates significance at 1%

Phenolic compounds, including anthocyanins, exhibit strong antioxidant activity and they have been shown in many studies to contribute significantly to the total antioxidant capacity of fruit samples.[1–3,5,9,11,23,26,29–32] Specifically, phenolic-rich fruits with highly pigmented flesh such as elderberries are often found to contain the highest antioxidant capacities. Among common small fruits, elderberries are perhaps most comparable to blackberries and black raspberries. Halvorsen *et al*.[33] listed blackberries as having the highest antioxidant contents per serving among the 1113 food samples, in National Food and Nutrient Analysis Program of the US Department of Agriculture. In other studies, dark-fleshed fruit typically displayed higher levels of antioxidants than their red-fruited counterparts.[26,27,31,32] Methodological differences make direct comparisons across the studies difficult. However, using standardized methods in our laboratory, the values of TP, TMA and antioxidant capacity among elderberry accessions resembled closely to those obtained for 19 black raspberry[34] and eight blackberry samples (data not shown) in companion studies.

Earlier studies of small fruit phytonutrient contents reported high correlations among TP and anthocyanin values and antioxidant capacities, as determined by oxygen radical absorbance capacity (ORAC) and FRAP assays.[3,29,31] We have uncovered similar strong relationships among accession TP and TMA levels and their associated antioxidant capacities, as determined by the FRAP (*r*^2^ = 0.84-0.85) and DPPH (*r*^2^ = 0.70-0.82) assays [Table 2]. Values of FRAP were lowest in Accession 13 (13.4 μmol TE/g fw) and highest in Accessions 5 and 10 (31.7 and 30.6 μmol TE/g fw, respectively), representing a difference that was approximately 2.3 fold. Patterns in antioxidant capacity as estimated by the DPPH radical were similar, except that values for Accession 5 were only in the mid-range.

Similar to our results, in other studies,[23,34] FRAP assay values were proportionally higher than DPPH assay values, presumably due to differences in reaction kinetics and steady state (absolute) antioxidant potentials of various reductive substrates as they interact with the two radicals. Moreover, the reactivity of an individual antioxidant in a given system was thought to depend upon the degree and nature of hydroxylation, methoxylation, acylation and or glycosylation associated with its structure, the chemistry of the radical with which it reacts and the chemical and physical environment in which the reaction takes place. Because antioxidant capacity estimates are likely to vary with techniques, the use of more than one antioxidant assay was recommended for the determination of antioxidant power of small fruit samples.[23] Although values from the two assay techniques were different, the data sets in this study were highly and significantly correlated [Table 2].

Levels of TA and TSS for each accession are also presented in Table 1 for a reference of sample maturity. Both the parameters were moderately variable among samples (CV = 19.1 and 14.2%, respectively). The relatively low soluble solid content of Accession 13 may have reflected a degree of immaturity in this sample, but the remainder of the values suggested that accessions were harvested at similar stages of ripening. Since maturity of fruits plays an important role in accumulation of antioxidant properties,[29] immature fruits may display lower antioxidant capacity. TA values were significantly and positively related to DPPH, TP and TMA values (*r* = 0.71, 0.60, 0.73, respectively; Table 2); TSS levels were not related to other parameters. The positive relationship between TA and other parameters was unlike to those found in ripening black and red raspberries and other fruits.[26,30,35] The positive relationship between TMA and TA is particularly surprising. In most small fruits, pigment accumulation continues and catabolism of titratable acids increases throughout the ripening process and during the storage of fruits. Conversely, unripe strawberry and/or blackberry fruit contained higher values for TP (presumably non-anthocyanin phenolics) or specific flavonoids than ripe fruit in studies by Wang and Lin[26] and Siriwoharn *et al*.,[36] which elevated sample antioxidant capacities. Phytochemical changes during American elderberry maturation have yet to be published. If undertaken, such studies may help to explain these phyisologically intriguing relationships while offering a clearer assessment of the phytonutritional consequences of harvesting fruit prior to its full ripeness.

In this study, we characterized the phenotypic variation phytonutrient content and antioxidant capacity among selected American elderberry accessions and determined the strength of relationships among commonly measured variables within the population. Sample TMA contents and their relative contribution to TP values were perhaps most variable among our accessions. Anthocyanins are routinely reported in a range of clinical and physiological studies to exhibit chemoprotective effects against an array of degenerative diseases of aging. Elderberry anthocyanins, specifically, have been shown to protect against oxidative stress in bovine aorta and porcine coronary artery endothelial cells.[14,16] In concert with other elderberry polyphenolic compounds, they promote quinone reductase (a protective enzyme) and inhibit cyclooxygenase-2 (COX-2) and ornithine decarboxylase (promotive enzymes) associated with the initiation and promotion phases of carcinogenesis.[13] The relative importance of these phenolic compounds to the health-promoting properties of elderberries justifies the development of cultivars with optimized levels of anthocyanins and other polyphenolic constituents.

## CONCLUSION

Lee and Finn[11] advocated American elderberry improvement using a traditional breeding approach. Among the 10 cultivars they examined, they encountered what they considered to be sufficient variability to begin crossing and selection program for developing elderberry breeding lines with high or low anthocyanin and phenolic levels. Herein, we found variability in phytonutrient content and antioxidant capacity among our 14 accessions to be very similar to that uncovered by Lee and Finn,[11] suggesting that similar breeding progress for phytonutrient content could be made using selections from the wild. As the germplasm pool of this species is essentially unexploited, the use of wild germplasm may offer an opportunity to incorporate a greater array of superior horticultural, post-harvest or processing traits into new or existing cultivars with superior phytonutrient profiles. In concordance with Lee and Finn,[11] our study is far too limited in scope to be definitive in its conclusions. The first step in a program to improve American elderberry would likely involve an extensive study of variability within the species across its native range. Moreover, although a divergent selection approach could be initiated with the variability at hand, medical and physiological evidence delineating effective target concentrations for specific compounds will be paramount if we are to optimize American elderberry fruit for human health.
